# Identification and Cultivation of Biotechnologically Relevant Microalgal and Cyanobacterial Species Isolated from Sečovlje Salt Pans, Slovenia

**DOI:** 10.3390/md24010026

**Published:** 2026-01-08

**Authors:** Eylem Atak, Petra Tavčar Verdev, Marko Petek, Anna Coll, Daniel Bosch, Marko Dolinar, Viktoriia Komarysta, Neli Glavaš, Ana Rotter

**Affiliations:** 1 Marine Biology Station Piran, National Institute of Biology, 6330 Piran, Slovenia; eylem.atak@nib.si (E.A.); daniel.bosch@nib.si (D.B.); 2 Department of Environmental Technologies, Jožef Stefan Institute, 1001 Ljubljana, Slovenia; 3 Faculty of Chemistry and Chemical Technology, University of Ljubljana, 1001 Ljubljana, Slovenia; petra.tavcarverdev@fkkt.uni-lj.si (P.T.V.); marko.dolinar@fkkt.uni-lj.si (M.D.); 4 Department of Biotechnology and Systems Biology, National Institute of Biology, 1001 Ljubljana, Slovenia; marko.petek@nib.si (M.P.); acoll@semillasfito.com (A.C.); 5 Department of Botany and Plant Ecology, V. N. Karazin Kharkiv National University, 61022 Kharkiv, Ukraine; v.p.komarysta@karazin.ua; 6 Institute of Sustainable Processes, University of Valladolid, 47080 Valladolid, Spain; 7 Department of Chemical Engineering and Environmental Technology, University of Valladolid, 47080 Valladolid, Spain; 8 SOLINE Pridelava Soli d.o.o, Portorož, 6330 Piran, Slovenia; neli.glavas@soline.si

**Keywords:** culture conditions, halophilic organisms, hypersaline environment, identification of microorganisms, shotgun metagenomics, photosynthetic microorganisms

## Abstract

Studies of complex natural environments often focus on either biodiversity or on isolating organisms with specific properties. In this study, we sought to widen this perspective and achieve both. In particular, hypersaline ecosystems, such as the Sečovlje salt pans (Slovenia), are particularly promising sources of novel bioactive compounds, as their microorganisms have evolved adaptations to desiccation and high light intensity stress. We applied shotgun metagenomics to assess microbial biodiversity under low- and high-salinity conditions, complemented by isolation and cultivation of photosynthetic microorganisms. Metagenomic analyses revealed major shifts in community composition with increasing salinity: halophilic Archaea became dominant, while bacterial abundance decreased. Eukaryotic assemblages also changed, with greater representation of salt-tolerant genera such as *Dunaliella* sp. Numerous additional microorganisms with biotechnological potential were identified. Samples from both petola and brine led to the isolation and cultivation of *Dunaliella* sp., *Tetradesmus obliquus*, *Tetraselmis* sp. and cyanobacteria *Phormidium* sp./*Sodalinema stali*, *Leptolyngbya* sp., and *Capilliphycus guerandensis*. The newly established cultures are the first collection from this hypersaline environment and provide a foundation for future biodiscovery, production optimization, and sustainable bioprocess development. The methods developed in this study constitute a Toolbox Solution that can be easily replicated in other habitats.

## 1. Introduction

Microalgae and cyanobacteria are photosynthetic microorganisms. The fundamental structural difference between them is that microalgae are eukaryotes that contain membrane-bound organelles such as a nucleus, mitochondria, and chloroplasts, whereas cyanobacteria are prokaryotes that lack membrane-bound organelles but possess non-membranous structures [1]. Both are widely found in aquatic and terrestrial ecosystems and can multiply rapidly using natural resources such as sunlight, carbon dioxide, and water. Due to these characteristics, they serve as a fundamental component of trophic networks and contribute significantly to the global carbon cycle [2].

Microalgae and cyanobacteria are attractive for biotechnological use due to their short generation times and the ability to grow on non-arable land [3]. Almost every newly discovered cyanobacterium produces one or several unique secondary metabolite (toxins, antibiotics, etc.) [4]. Microalgae are particularly rich in antioxidant pigments, especially carotenoids, which include both carotenes (e.g., carotenes such as α-carotene and β-carotene) and xanthophylls such as lutein and zeaxanthin. These pigments reduce oxidative stress by providing protection against free radicals at the cellular level and contribute to the balance of biological systems [5]. In addition, microalgae contain valuable lipids such as fucosterol and β-sitosterol, as well as polysaccharides. Both microalgae and cyanobacteria produce many industrially relevant bioactive compounds such as polyphenols, phytosterols, tocopherols, polyunsaturated fatty acids (PUFAs), and polysaccharides [6,7]. These molecules exhibit a wide range of biological activities, including antitumor, antibacterial, antifungal, antiviral, anticoagulant, antihypertensive, antioxidant, anti-inflammatory, and cytotoxic ones [8]. Their retinal proteins, pigments, extracellular polysaccharides and other metabolites are already being used in medical, cosmetics, food/feed, materials, bioremediation, and other industries [3]. 

The exact number of algae and cyanobacteria species found in nature is still unknown. Nevertheless, estimates indicate that there are more than 70,000 species, of which more than 40,000 have been scientifically identified. This vast biological diversity is noteworthy not only for their roles in ecosystems but also for their economic significance [9]. Indeed, the global market value of products derived from microalgae and cyanobacteria has been steadily increasing in recent years [10]. The microalgae market was reported to be worth approximately USD 3.4 billion in 2020, and this value is expected to reach USD 19.85 billion by 2028, continuing to grow at a rate of 7.8% [1,11]. Among the main reasons for this increase are the recognition of the potential of valuable compounds contained in microalgae and their evaluation as a sustainable alternative in food and energy production [12]. In this context, the genera *Dunaliella*, *Botryococcus*, *Chlamydomonas* and *Chlorella* are the most frequently used microalgae for commercial industrial applications [13]. The market size of cyanobacteria-based biological products was reported to be approximately USD 348 million in 2020, showing an average annual growth rate of 10.5% [11]. Among cyanobacteria, *Arthrospira* (*Spirulina*) stands out in particular. The global *Arthrospira* (*Spirulina*) production has approximately doubled in the last two decades, currently estimated to be at least 30,000 tons yearly [14]. Overall, these numbers demonstrate the growing importance of microalgae and cyanobacteria on a global scale, both in terms of biological diversity and economic potential [1]. Despite this potential, cyanobacteria and microalgae have found only limited industrial use to date, which highlights their significant market growth potential [15].

Beyond their general biotechnological potential, increasing attention has recently been directed toward microorganisms that thrive under extreme environmental conditions. Indeed, some microorganisms can survive in environments with high or low pH (acidophiles and alkaliphiles), extreme temperatures (thermophiles and psychrophiles), high pressure (piezophiles), high salinity (halophiles), and even multiple stress factors (polyextremophiles) thanks to their extraordinary adaptation mechanisms [16]. Microorganisms (prokaryotes and eukaryotes) living in extreme environments can produce extremozymes and extremolytes, the latter being unique organic compounds that do not play a direct role in the normal growth, development, or reproduction processes [17,18]. Under environmental stress conditions, such as low temperature, high salinity, and nutrient limitation, the accumulation of omega-3 fatty acids increases and is considered an adaptive mechanism to maintain cell membrane fluidity. *Nannochloropsis* species can contain up to 37.8% eicosapentaenoic acid (EPA), an omega-3 PUFA. Certain environmental conditions, for example, low temperature and high light intensity, increase EPA production in *Nannochloropsis*, while phosphate limitation and elevated CO_2_ levels have similar effect in *Phaeodactylum tricornutum* [19]. *Spirulina platensis*, an alkaliphilic cyanobacterium, has a higher vitamin B12 content than any other plant or animal source. It is also an excellent source of provitamin A, vitamin E, thiamine, biotin, inositol, and cobalamin [20]. These stress-driven metabolic responses highlight the potential of extremophilic microalgae and cyanobacteria as targeted platforms for optimized biotechnological applications. 

Advances in culturing methods under laboratory conditions and genetic and metabolic data by omics approaches have contributed to a better understanding of the ecological roles and evolutionary processes of extremophiles. The enzymes and metabolites produced by these organisms emerge as promising resources for biotechnology, drug development, bioremediation, energy production, and sustainable industrial applications. All these developments reveal that extremophiles are a critical area of research not only for understanding biodiversity in nature but also for future scientific and technological advances [21].

One of the extreme environments of increasing interest are ecosystems with high or extreme salinity, which host diverse microbial communities. The molecular adaptations and metabolic pathways of microorganisms living in halophilic environments suggest promising biotechnological applications [22,23,24]. Coastal lagoons in Portugal and Spain, the Camargue delta in France, the salt flats of Italy, and the saline wetlands of the Balkan Peninsula represent some of the most characteristic hypersaline systems in Europe in terms of carotenoid-producing microalgae and halophilic microorganisms. [24]. There is a continuous need to understand the metabolic pathways of extremophilic or extremotolerant organisms and the environmental influences that trigger the production of valuable compounds that can enable the marine bioprospecting approach that encompasses sampling, determination of biological diversity, isolation of cultures, screening for new metabolites, isolation and testing of target metabolites, and development of commercial products [25,26]. The isolation and cultivation of organisms that produce biotechnologically relevant compounds remains a critical milestone in this pipeline [27].

Within this research, we conducted a comprehensive assessment of the biological diversity of the Sečovlje salt pans in Slovenia. These extreme ecosystems, which have not yet been fully researched, offer untapped potential from a biotechnological perspective. In line with this, our study contributes to the bioprospecting pipeline development by identifying and culturing biotechnologically prospective microorganisms.

## 2. Results

### 2.1. Metagenomic Analysis of Petola

A total of 21 petola samples were collected between April and September 2023 (Table 5), capturing the seasonal variability in temperature and salinity, which we expected to be the most likely drivers of the observed changes in microbial composition. Low salinity refers to values between 4.4 and 5.1% S (April, May, September), whereas high salinity refers to values between 26.8 and 28.7% S (July and August) (see Table 5).

**Figure 1:**
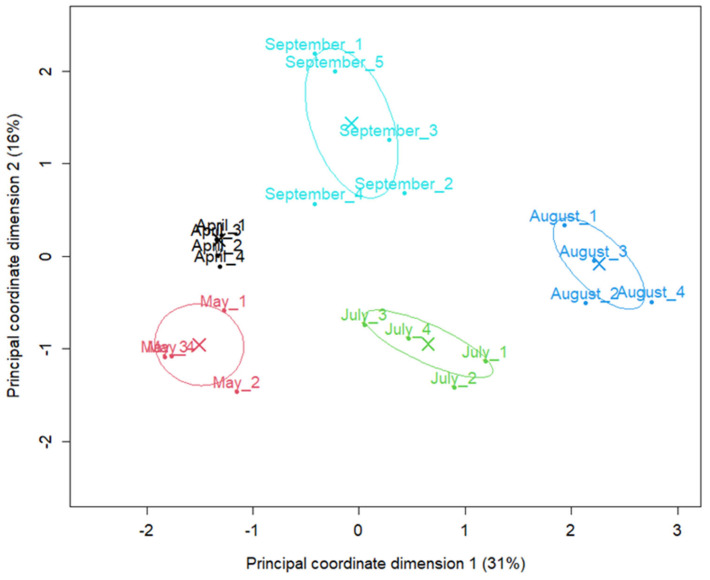
Abundance-based ordination of shotgun metagenomic samples by sampling month. Samples were colored and labeled by sampling month. Principal coordinate (MDS/PCoA) plot of samples based on TMM-normalized species count data, generated with the limma plotMDS function. Distances between points represent the leading log2-fold-change in species abundances between samples. Samples are colored and labelled by sampling month; crosses (X) indicate monthly centroids and ellipses the corresponding 95% confidence intervals. The percentages in parentheses on the axes indicate the proportion of between-sample variation captured by each dimension.

The seasonal shift in abundance of microbial species was evident in the multidimensional scaling (MDS) plot, where samples cluster distinctly by month with August forming a particularly distinct cluster (Figure 1). The first axis (Principal coordinate dimension (1) accounted for 31% of the total variation (dissimilarity), whereas the second axis (Principal coordinate dimension (2) accounted for 16%. Together, these two axes accounted for 47% of the total variation, with samples from different months located in markedly different regions. Samples from April were located very close to each other, suggesting very similar abundance profiles among replicates. In contrast, samples from May, July, August and September showed a wider distribution, suggesting time-dependent changes in taxon abundance.

**Figure 2:**
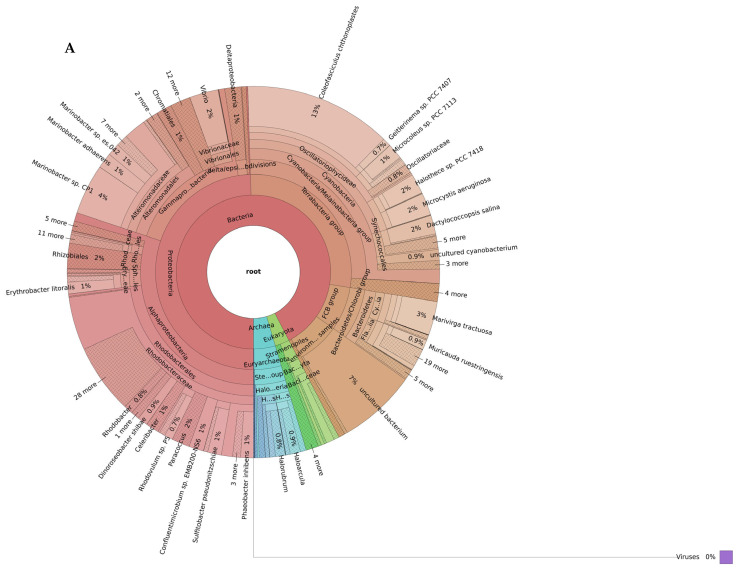

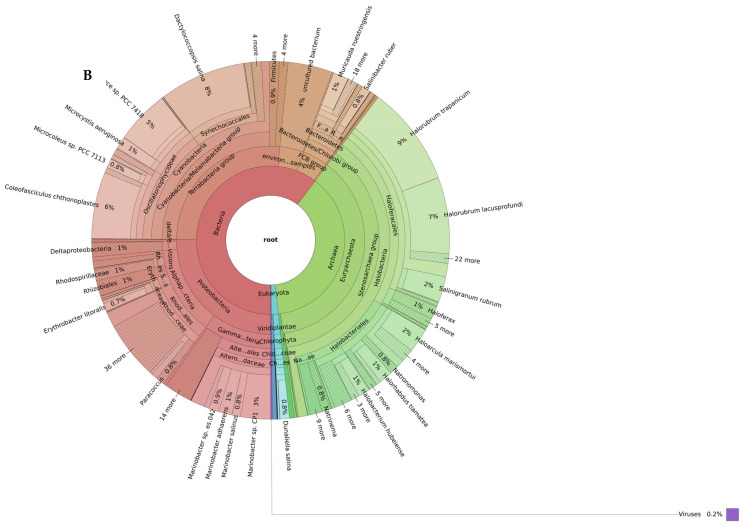
Biodiversity of the Sečovlje salt pans petola mat at low (**A**) and high (**B**) salinity levels. Only the most abundant phyla present in all samples (shared_summary) are shown in the outermost ring of each plot (bacteria in red, Archaea in green and eukaryotes in blue). Interactive Krona plots generated by recentrifuge are available at github.com/NIB-SI/AlKoSol. Legend: Taxonomic assignments shown in this Figure 2 reflect the nomenclature present in the Centrifuge default database (built in 2018), which follows earlier taxonomic classifications. Updated taxonomy: Firmicutes (Bacillota), Actinobacteria (Actinomycetota/Actinobacteriota), Bacteroidetes (Bacteroidota), Proteobacteria (Pseudomonadota), and Chlorobi (now class Chlorobia within Bacteroidota) based on Genom Taxonomy Data Base.

The shotgun metagenomic taxonomic read classification showed that the petola was inhabited by a rich microbial community dominated by Bacteria, followed by Archaea and to a smaller extent eukaryotes (Figure 2).

#### 2.1.1. Low-Salinity Biodiversity

At low-salinity conditions, bacteria constituted the vast majority of microbial communities, representing approximately 86% of total diversity (Figure 2A). In this environment, cyanobacteria (26%) and Rhodobacterales (23%) belonging to Alphaproteobacteria stand out as the dominant taxa. In addition, Alteromonadales (11%), Vibrionales (3%), and Chromatiales (1%) from the Gammaproteobacteria subphyla were found in significant proportions. Furthermore, phyla such as Actinobacteriota (7%) and Bacteroidota (7%) also constituted a significant portion of the community. Archaeal communities were represented at a limited level in the low-salinity environment. In particular, the Halobacteria group reached only 4%. Eukaryota members were identified as only a small fraction (2–3%).

Among the identified species in the low-salinity petola, species with biotechnological potential are listed in Table 1.

#### 2.1.2. High-Salinity Biodiversity

Organisms in the petola mat samples taken in high-salinity conditions consisted of 56% bacteria, 37% Archaea, 2% eukaryotes (Figure 2B). The bacterial domain included representatives from Alphaproteobacteria (57 species), Bacteroidota (48 species), Gammaproteobacteria (36 species), cyanobacteria (31 species), Bacillota, Actinobacteriota and uncultured subgroups. Among the species in the high-salinity petola, some species with biotechnological potential are listed in Table 2.

These species were observed in all petola mat samples taken under high-salinity environmental conditions. Metagenomic results showed that the Archaeon *Halorubrum trapanicum* was the most common species representing 9% of all species in petola mat samples with high salinity.

### 2.2. Influence of Salinity on Microbiome Composition

Higher salinity significantly influenced the composition of the microbial community. The most pronounced shift was observed in Archaea, which accounted for only 5% of the community under low-salinity conditions but increased to 38% under high-salinity conditions (Figure 2). Within Archaea, the halotolerant genus *Halorubrum* increased in abundance from 19% at low salinity to 47% at high-salinity conditions. 

Even more strikingly, within Eukaryota, elevated salinity conditions strongly favored the proliferation of the unicellular green algae *Dunaliella salina*, which represented 0.3% of Eukaryota in low-salinity samples but rose to 54% under high-salinity conditions (Figure 2, see Supplementary Table S2—Petola species read classification). Among Stramenopiles, *Nannochloropsis* showed the greatest difference between salinity levels, maintaining a relative abundance of about 9–10% of the Eukaryotic community in both environments (see Supplementary Table S2—Petola species read classification).

In both salinity conditions, cyanobacteria were present at similar levels (25–26%), with the community dominated by *Coleofasciculus chthonoplastes*, followed by *Dactylococcopsis salina*, *Halothece* sp. PCC 7418, and *Microcystis aeruginosa*.

Of the 110 robustly differentially abundant species, 57 were more abundant and 53 were less abundant in the high-salinity samples compared with low-salinity samples (Supplementary Table S3—table merged_results_Brine_salinity_factor). The largest increase in abundance was observed for *Dunaliella salina*, followed by several halotolerant Archaea and Bacteria. Among Archaea, several *Halorubrum* species exhibited a 30- to 80-fold increase in abundance relative to low salinity. A more than 60-fold increase was also detected for the bacterium *Halanaerobacter jeridensis.* Species that were less abundant in high-salinity samples predominantly belong to Alphaproteobacteria and several marine eukaryotes. Notably, none of the Archaeal species decreased in abundance under the high-salinity conditions.

### 2.3. Cultivation, Isolation and Identification of Cultures

Several photosynthetic species capable of tolerating high-salinity conditions, such as those present in salt pans, were detected in our samples. These species were successfully isolated and cultivated under laboratory conditions. By applying various isolation and purification techniques (such as streak plate method, single filament transfer, serial dilution, and single colony transfer) and cultivation in growth media with varying salinity levels (mimicking freshwater, marine, and hypersaline conditions), we managed to cultivate different photosynthetic microorganisms from Sečovlje salt pans and analyzed them by DNA barcoding of the internal transcribed spacer (ITS) genomic region (ITS1-5.8S rDNA-ITS2 for green algae, and16S-23S rDNA ITS for cyanobacteria) (Table 3).

**Figure 3:**
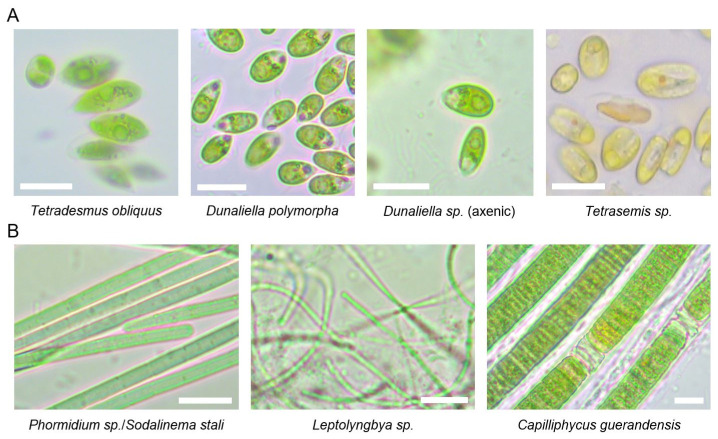
Microscopy images of algal (**A**) and cyanobacterial (**B**) species isolated from Sečovlje salt pans. Representative cells were imaged by Primo star (Zeiss). Scale bar corresponds to 10 μm.

We obtained both axenic and mixed cultures of different *Dunaliella* species, a mixed culture of *Tetraselmis* sp. along with an unidentified species (two PCR-products were observed with AGE analysis; however, direct sequencing revealed that one did not match any known species), and an axenic culture of the freshwater alga *Tetradesmus obliquus*. Only one cyanobacterial culture was purified to axenicity, identified as the filamentous cyanobacterium *Phormidium* sp./*Sodalinema stali*. Additionally, two mixed cultures containing at least two different cyanobacteria were cultivated under laboratory conditions (*Leptolyngbya* sp. co-cultured with Prochlorotrichaceae bacterium, and *Capilliphycus guerandensis* co-cultured with an unidentified cyanobacterium). These cultures could not be purified to axenicity during several months of the experiment. Axenicity was determined by microscopic examination and the AGE-analysis of genomic markers (algal ITS1-5.8S rDNA-ITS2 or bacterial ITS) amplified using universal primers. Culture was marked as axenic if a single band was recorded in the AGE-analysis of PCR-products, and no visible contaminants were observed macro- and microscopically. Light microscopy was used to confirm DNA barcoding results (Figure 3) and to monitor cell culture purity.

A total of 23 laboratory cultures were obtained from the collected petola and brine samples (Table 5). Using the DNA barcoding approach, we were able to identify different microalgae and/or cyanobacteria in these cultures (Table 3). However, in approximately 15% of cultures, the microalgae/cyanobacteria species could not be identified using DNA barcoding, due to different factors: difficulties with culture growth, failure of universal primers used for DNA barcoding to produce PCR products, unsuccessful sequencing of the PCR amplicons (corresponding to algal or bacterial ITS regions), or the absence of matching reference sequences in GenBank. Interestingly, analysis of two laboratory cultures revealed that they contained the ciliate *Schmidingerothrix salina*. In both cases, this organism was detected in culture originating from brine samples that were collected during the high-salinity period (August 2023).

## 3. Discussion

This study evaluated the diversity of microorganisms inhabiting the Sečovlje salt pans in Slovenia and established laboratory cultures, which forms a basis for the extraction of high-value-added products produced by microalgae and other microorganisms that can survive in hypersaline environments. These organisms are of both scientific and industrial importance due to their adaptability to extreme environmental conditions. Although a part of microbial diversity is maintained in several global microbial culture collections, sourcing biotechnologically relevant organisms locally, even from natural parks, is an important market value proposition. Indeed, the increase in consumer awareness to purchase and use sustainably sourced, local and safe products is driving the development of several industrial sectors, such as food, feed and cosmetics [67,68,69]. Local food and other industrial systems can generate higher margins for producers, higher-paid employment opportunities, and increased local economic multipliers compared against ‘long’ supply chains [70].

First, metagenomics was used to assess the seasonal variation in microbial diversity in the Sečovlje salt pans in Slovenia. To our knowledge, there are no studies evaluating the microbial diversity in these salt ponds by focusing on species with biotechnological potential. This is a critical step towards targeted approaches of isolation and potential applications in various industrial sectors. Indeed, Sečovlje salt pans contain species (such as *Dunaliella salina*) already used in industrial applications which have not been directly targeted for the local industries.

Second, we isolated and cultivated green microalgae and cyanobacteria under laboratory conditions with the primary aim of obtaining axenic cultures of hypersaline photosynthetic microorganisms, originating from the Sečovlje salt pans in Slovenia.

For many industrial purposes, obtaining axenic (pure) cultures is crucial. These pure cultures can then be characterized in terms of their taxonomy, biochemical and physiological properties, and biotechnological potential. Axenic cultures of biotechnologically promising species can be cultivated in large-scale systems for biomass production or used for further metabolic engineering studies [3,10,71]. An additional practical advantage of halophiles is that their cultivation in high-salt environments naturally suppresses contaminants, thereby reducing the need for sterile conditions and lowering sterilization costs [72,73].

Although culture-independent molecular methods have revolutionized our understanding of microbial diversity, culturing remains indispensable. This is because only cultured microorganisms can be used to perform controlled physiological tests, produce new biomolecules, facilitate transition to industrial applications (e.g., enzyme, antibiotic, and pigment production), and enable taxonomic characterization of new species. As a result, a comprehensive approach that begins with metagenomic analysis and continues with targeted culturing emerges as the most effective strategy for both enhancing our fundamental microbiology knowledge and making environmentally or biotechnologically valuable microorganisms available for use.

### 3.1. Microbial Biodiversity of the Sečovlje Salt Pan

To date, only a limited number of biodiversity studies have been conducted in the Sečovlje salt pans, particularly in terms of taxonomic resolution. This research is the first to focus on taxonomic resolution at the species/genus level and to place special emphasis on species with biotechnological potential. Furthermore, it should be noted that most previous shotgun biodiversity studies of salt pans have focused more on bacteria, Archaea, and viruses, with less emphasis on eukaryotes [74,75,76,77,78]. This may be due to the relatively low abundance of eukaryotes; however, it also presents an important opportunity for further research [79,80].

A pivotal bacterial diversity study of the Sečovlje petola in contrasting salinity levels was performed by Tkavc and colleagues using a low-throughput 16S cloning and Sanger sequencing approach. Our shotgun metagenomics profile results closely resemble the active petola’s top layer described before, showing similar proportions of Gammaproteobacteria, cyanobacteria, Betaproteobacteriota, Bacteroidota, and Bacillota [78].

In the higher salinity, [75] observed higher abundance of Bacillota of the order Halanaerobiales, Actinobacteriota of the genera *Ilumatobacter* and *Nitriliruptor*, and halophilic Alphaproteobacteria as well as reduced abundance of Planctomycetota [75]. Comparison with our DNA results also revealed an increase in Bacillota, primarily attributable to halophilic species such as *Halanaerobacter jeridensis*, *Halobacteroides halobius*, and *Acetohalobium arabaticum* (see Supplementary Table S3–merged_results_Brine_salinity_factor). In our study, only about 1% of the reads were classified as Actinobacteriota. Compared to approximately 6% reported by [75], we did not observe any change in the abundance of this phylum between low- and high-salinity conditions. Within Actinobacteriota, approximately half of the reads were classified as the species *Ilumatobacter coccineus*; however, we did not observe any shotgun reads that were classified as *Nitriliruptor*. Within Alphaproteobacteria, our results indicate that only two low-abundance species, *Rhodovulum* sp. JZ3A21 and *Sphingopyxis macrogoltabida*, were able to proliferate under high-salinity conditions, whereas the abundances of 24 other Alphaproteobacteria were reduced. Similarly, the Planctomycetota were present in very low amounts (<1% of bacteria) in our samples and none of the PVC (Planctomycetota, Verrucomicrobiota and Chlamydiota) bacterial group taxa showed a difference in abundance between high and low salinities. Contrary to 36–54% of cyanobacteria belonging to Leptolyngbyaceae reported by Glavaš and colleagues, this family was represented on average by less than 1% of reads in our samples (Supplementary Table S2—Petola species read classification) [75].

Our findings reveal that salinity is a dominant driver of microbial community structure in petola, consistent with studies of other salt pans such as those along the Mississippi Gulf Coast [81]. Under low-salinity conditions (Figure 2A), we found that the community was dominated by Pseudomonadota, Bacteroidota and Planctomycetota, with *Pseudomonas* and other facultative anaerobes. In contrast, at high salinity (Figure 2B), the community shifted significantly toward Archaea, with halophilic Halobacteriaceae and Haloferacaceae in the surface layers. High salinity therefore imposes strong selective pressure that excludes non-halophilic groups such as Planctomycetota and reduces the overall bacterial diversity [81].

Sečovlje salt pans contain several eukaryotic species of biotechnological importance. *Fistulifera solaris* stands out for its high capacity to produce PUFAs, particularly EPA. EPA is an essential fatty acid for human health, with functions including cardiovascular protection, anti-inflammatory effects, and support for brain health. Therefore, the lipids produced by this species are considered valuable in the field of dietary supplements and functional foods, and also show promise for pharmaceutical applications [39,40]. Similarly, carotenoids such as diadinoxanthin and fucoxanthin synthesized by *Cylindrotheca closterium* are gaining attention due to their potent antioxidant properties. Fucoxanthin is used in the food and cosmeceutical industries, particularly for its potential effects against obesity and its photoprotective properties. The carotenoid profile of this species contributes to the diversification of natural antioxidant sources and offers a sustainable alternative to synthetic antioxidants [41,42,43]. Diatom species such as *Thalassiosira oceanica* are important not only for metabolite production but also for structural biomaterials. This species capacity to produce bio-silica is of interest for nanotechnology and biomedical applications. In addition, its antimicrobial properties offer potential applications in the medical field. Bio-silica-based materials are being evaluated in innovative biomedical applications such as tissue engineering and drug delivery systems [44].

Other biotechnologically relevant species found in Sečovlje salt pans include *Durinskia baltica* from the Dinoflagellate class, *Lotharella oceanica* from the Protozoa class, and *Emiliania huxleyi*, a Coccolithophore, which all play a major role in the production of calcium carbonate [82]. Finally, our results revealed the presence of nitrogen (N_2_)-fixing *Halothece* sp. [83], *Phormidium lucidum* with rich carbohydrate, phycobiliprotein, and vitamin C content [84] and *Pseudanabaena* sp. with potential for biodiesel production [85]. 

Research on microbial diversity in saline ecosystems has expanded substantially over the past decade, providing a clearer understanding of both the ecological roles and biotechnological potential of extremophilic phototrophs. When comparing salt lakes and ponds in different geographical regions, the composition and dominant groups of microbial communities vary depending on environmental conditions. The study on the Monegros lakes revealed that inland salt lakes possess high phylogenetic richness and unexpected levels of diversity, particularly among protists, and exhibit a high degree of genetic novelty in Archaea (especially in the Halobacteriaceae and DHVEG-6 groups (Deep Hydrothermal Vent Euryarchaeotal Group 6)) [86]. In contrast, at Sečovlje Salt pans, cyanobacteria and phototrophic Pseudomonadota (Rhodobacterales) were dominant under low-salinity conditions, while Archaea (Halobacteria) communities were prominent at high salinity. Therefore, the role of Archaea became more pronounced with increasing salinity in both systems. As salinity increases, the dominance of Archaea, particularly members of the class Halobacteria, becomes evident, representing a characteristic pattern widely described in the literature for hypersaline ecosystems [87]. This transition indicates that extreme salinity conditions are tolerated by Archaea with exceptionally high osmotic stress resistance, and that these groups achieve ecological advantage in such environments through metabolic strategies based on phototrophic pigments and retinal proteins [24]

### 3.2. Targeted Organisms with Biotechnological Potential: Isolation of Microalgal and Cyanobacterial Cultures

Salt pans are areas where light, temperature and salinity conditions change rapidly, and these environmental changes affect microbial diversity and the content of the bioactive substances that microorganisms inhabiting salt pans produce. Consequently, abiotic parameters such as light transmission and oxygen availability significantly affect culture conditions, thereby influencing both microbial growth and the production of target compounds [88]. For example, *Dunaliella* species have higher antimicrobial properties under osmotic stress conditions [89]. Therefore, a better understanding of the chemical and physical conditions, and their effect on algal metabolism is crucial for laboratory-scale production and future industrial scale-up. The initial understanding of the environmental effects, such as temperature and salinity throughout the year, can thus contribute to improving the cultivation conditions for optimal yields of biomass and targeted bioactive compounds.

Through our targeted cultivation attempts, we aimed to establish cultures of species with potential for their use in various industries (Table 4).

Through regular sampling campaigns on the same location, our study demonstrates that the microbial community changes with increased salinity and temperatures from April to August (Figure 2). The most striking is the abundance increase in the green algae *Dunaliella salina* and the halophilic Archaea. These are typical representatives of hypersaline environments such as solar salterns, brine pools, salt lakes, deserts and intertidal zones [110].

We successfully cultivated axenic and mixed cultures of green microalgae from the genus *Dunaliella* (Table 3). Different species of *Dunaliella* were obtained from samples collected during both high- and low-salinity seasons, indicating that these algae are present in the Sečovlje salt pans throughout the whole year. They can be efficiently isolated using hypersaline nutrient media, such as Modified Johnson’s medium and Artari medium (see Supplementary Table S1—Culture_Medium_Contents). This finding aligns with metagenomics results from this study, which detected multiple *Dunaliella* species in petola samples in low- and high-salinity season (see Figure 2, see Supplementary Table S3—merged_results_Brine_salinity_factor). Indeed, besides *D. salina*, which is one of the most halophilic species within the *Dunaliella* genus, other less halophilic species also inhabit the salterns. For example, *D. viridis* and *D. parva* growth is superior in mediums with intermediate salinity (around 1 M NaCl), while *D. salina* thrives optimally at higher salinities—up to 3 M NaCl [111].

The taxonomic classification of the isolated *Dunaliella* species based on morphological traits and DNA barcoding of the ITS region proved challenging. Earlier studies relied heavily on morphological features; however, because cell size and shape change considerably in response to environmental conditions such as nutrient availability, salinity, temperature, and light intensity, morphology-based identification alone does not allow reliable species-level resolution [111,112]. Therefore, modern approaches use molecular techniques like DNA barcoding of the 18S rDNA and ITS regions, analysis of ITS2 secondary structure, phylogenetic reconstruction, intron analysis of the 18S rDNA, and fingerprinting methods for accurate species identification [111,113,114]. The ITS region is particularly effective for barcoding members of Chlorophyta group, including *Dunaliella*, due to its high intraspecies variability, availability of universal set of primers, and well-established database of reference sequences [115]. Despite the effectiveness of ITS barcoding for many green algae, this study identified only *Dunaliella polymorpha* to the species level. For other samples, species-level identification was not achieved due to high similarity among the sequenced regions and the presence of multiple similar reference sequences in GenBank. Previous reports have highlighted issues with having confusing and potentially incorrect taxonomic classifications for *Dunaliella* species. Notably, one of the main challenges in *Dunaliella* taxonomy is the absence of a well-established and universally accepted classification system. Both *Dunaliella viridis* and *Dunaliella salina* have been shown to consist of multiple genetically distinct clades rather than forming single coherent phylogenetic groups [116,117,118]. It is increasingly apparent that many database entries, particularly from older studies, are mislabeled or misnamed, indicating a need for a revision of *Dunaliella* taxonomy [111,117]. Importantly, incorporating ITS2 secondary structure analysis may overcome some of these limitations. Structural features such as compensatory base changes and helix architecture provide phylogenetically informative characters that are not evident from primary sequence alone. Detailed ITS2 structural assessment has already proven useful in resolving complex species boundaries, as demonstrated in the taxonomic re-evaluation of *D. viridis* [114].

Besides *Dunaliella* species, two other green microalgae were isolated: *Tetraselmis* sp. and *Tetradesmus obliquus*. Both hold high potential for biotechnology use [90]. For example, *Tetraselmis* sp. can be an alternative food production source [119]. Indeed, *Tetraselmis* sp. is rich in vitamins, carotenoids and protein, similar to microalgae such as *Chlorella* and *Arthrospira,* which are considered as safe food that does not contain pathogens and toxic components [119]. *Tetradesmus obliquus* produces lutein, β-carotene, protein and essential fatty acids used in cosmetics, medicine and pharmaceuticals [120]. Interestingly, despite its laboratory growth, the genus *Tetradesmus* was not identified in the metagenomics analysis of petola samples, and *Tetraselmis* was present only in some of them at a low relative abundance. This indicates that these species might inhabit the brine but not the petola mat.

Next, we established cultures of various filamentous cyanobacteria. Species that grew in hypersaline nutrient media included *Phormidium* sp./*Sodalinema stali* (axenic culture) and *Leptolyngbya* sp. in combination with a Prochlorotrichaceae bacterium (mixed culture) (see Table 3). Additionally, cultivation in marine medium ASN-III (see Supplementary Table S1—Culture_Medium_Contents) enabled the growth of a mixed culture of *Capilliphycus guerandensis* and another filamentous cyanobacterium, likely from the order Leptolyngbyales, based on observed morphological characteristics (Figure 3).

Surprisingly, *Coleofasciculus chtonoplastes*, the predominant cyanobacterium identified by metagenomic analysis, could not be cultivated under laboratory conditions. This could be due to the lack of its adaptation to the laboratory conditions or a slower growth compared to other cyanobacterial strains (such as *Phormidium* sp./*Sodalinema stali*) under chosen conditions, which allowed the latter to outcompete all species present in the crude environmental samples. *C. chtonoplastes* is a mat-forming cyanobacterium [121]. The biotechnological potential has already been elucidated for mat-forming taxa, with their enzymes and antimicrobials [122]. Hence, the isolation of this species should remain a target in future studies.

The cyanobacterium *Phormidium* sp. is another species of biotechnological potential. It produces phycocyanin, which is important for cosmetics and medicine applications [123]. Many cyanobacterial species form extracellular mucous sheaths that are inhabited by heterotrophic microorganisms, such as bacteria and fungi [124]. Indeed, *Phormidium* sp. forms dense biofilms under high-salinity conditions as it has the capacity to produce significant amounts of extracellular polysaccharides (EPS) [125,126]. As filamentous cyanobacteria typically coexist within complex microbial mats in their natural environments, separation of species is challenging. Moreover, cyanobacteria are known to form symbiotic interactions with other microorganisms [127], which may explain why mixed cultures are often obtained as the final culture. The ability to grow mixed or axenic cultures was dependent on the salinity of the culture medium. While the detailed results are presented in Section 2, it is notable that hypersaline conditions favored the growth of axenic *Phormidium* sp./*Sodalinema stali*, whereas intermediate salinity supported more complex microbial associations [128,129]. Gradually increasing the salinity of the medium may be a promising strategy for purifying cyanobacterial hypersaline species.

## 4. Materials and Methods

### 4.1. Description of the Study Area

Located in southwestern Slovenia and covering an area of about 670 hectares, the Sečovlje Salina Nature Park is the largest coastal wetland in Slovenia and contains the northernmost active solar salt pans in the Mediterranean. The traditional manual salt harvesting is based on the cultivation of the microbial mat named petola, which covers the bottom of the crystallization pans. This active biological layer has many roles, like stabilizing the sediment surface, preventing contamination of the harvested salt with the bottom mud, and enhancing salt purity [74,75,77]. Recently, it was also revealed that salt from Sečovlje salt pans contains higher concentrations of glutamic acid, a key contributor to umami taste [130].

### 4.2. Sample Site

Samples of the microbial mat petola were collected from the crystallizing pan number S10 (45°29′21.58″ N, 13°35′59.17″ E) during spring, summer and autumn in 2023 (Table 5). Short vertical cores of the upper 1 cm were collected in plastic sterile containers. Brine salinity was measured with a hydrometer, and the temperature-corrected Baumé [131] was converted into mass percent salinity (*S* %) (Equation) [132,133]. In solar salt pans, the salinity of brine in the crystallizing pans is influenced by the evaporation of seawater for salt production. The cultivation of the microbial mat petola requires it to be predominantly covered by water of seawater salinity (approx. 4–5% *S*) during the pre-harvest (spring) and post-harvest (autumn) period. In contrast, during the summer season, the salinity of the brine increases significantly, up to 29% *S*. Low salinity refers to values between 4.4 and 5.1% *S*, whereas high salinity refers to values between 26.8 and 28.7% *S* (Table 5).

(1) $$S\%=-0.10537+1.107651\;\left({}^{\circ}\mathrm{Be}\right)-0.036102{\left({}^{\circ}\mathrm{Be}\right)}^{2}+0.002598{\left({}^{\circ}\mathrm{Be}\right)}^{3}-0.000052{\left({}^{\circ}\mathrm{Be}\right)}^{4}$$

### 4.3. Shotgun Metagenomics

#### 4.3.1. DNA Extraction and Sequencing

Genomic DNA was isolated from the petola samples using the PowerLyzer PowerSoil DNA Isolation Kit (Mo Bio Laboratories Inc., Carlsbad, CA, USA; Cat no. 12855-50). The isolation process was performed according to the manufacturer’s instructions, with minor modifications as follows: (i) Sample homogenization was performed using FastPrep (MP Biomedicals, Irvine, CA, USA) bead grinder, consisting of two homogenization rounds at 6.5 m/s for 45 s each, with an intermediate incubation period of 10 min at 70 °C accompanied by shaking at 2000 rpm. (ii) Following the precipitation with Solution C3, only 625 μL of the supernatant was transferred, and 1000 μL of Solution C4 was added in the subsequent step. (iii) The final elution of DNA was performed twice, each time using 75 μL of Solution C6 with a 10 min incubation. DNA was checked for purity and quantity on both NanoDrop spectrophotometer (Thermo Fisher, Waltham, MA, USA) and TapeStation (Agilent, Santa Clara, CA, USA). Samples passing the quality and quantity requirements of the sequencing service (A260/280 > 1.8 and concentration > 10 ng/μL) were selected for shotgun metagenomics sequencing (Table 5). DNA library preparation and sequencing on Illumina NovaSeq X Plus in paired-end 150 bp mode were performed by Novogene Ltd. (Sacramento, CA, USA).

#### 4.3.2. NGS Data Analysis

Adapter trimming and read quality control was performed by Novogene Ltd. Taxonomic classification of reads was performed using centrifuge v1.0.4 [134] by defining a random generator seed (−seed 42) and ensuring a single call per read (−k 1). Centrifuge-supplied “NCBI nucleotide nt” database built in 2018 was used. Because phylum-level nomenclature has been revised since that release, the names in the text were updated to current names, while the original Centrifuge labels are given in parentheses where relevant. Read counts obtained by centrifuge were further processed with Recentrifuged v1.14.0 [135] by setting parameters to retain only high-confident classifications (−−minscore 75) and removing any contaminants associated with taxa “Sinsheimervirus”, “chordates”, “unclassified sequences”, and “other sequences” (−x 1,910,954 −x 7711 −x 12,908 −x 28,384). Recentrifuge was run independently for low- and high-salinity samples. Interactive Krona plots were exported and are available at github.com/NIB-SI/AlKoSol.

Differential abundance analysis for identified species was performed in the R statistical environment v4.3.1 using MaAslin2 (Microbiome Multivariable Association with Linear Models 2) package [136]. The raw count data was filtered to retain only species with at least 2 reads in at least 10% of samples. For statistical comparisons, salinity level was used as a fixed effect variable, with “low” as the reference value (Table 5). To obtain a robust set of differentially abundant species, the intersection of three statistical methods was used: (i) linear model fit (LM) method, (ii) negative binomial (NEGBIN) method available in MaAslin2, and (iii) voomLmFit method from the edgeR package [137]. For the LM method, the count data was normalized with cumulative sum scaling (CSS) followed by log2 transformation, whereas for the NEGBIN method, CSS normalization was applied without additional transformation. For the voomLmFit method, the trimmed mean of M-values (TMM) normalization was used. Species were considered differentially abundant if they were detected in at least 12 out of 21 (57%) samples (as determined by MaAslin2 methods) and if statistical tests of all three methods yielded adjusted p-values below 0.05. The Principal Coordinate Analysis (PCoA) plot was generated using plotMDS function from the limma package and generated from filtered TMM-normalized count matrix as input.

### 4.4. Isolation and Maintenance of Microalgal/Cyanobacterial Cultures

A 1 × 1 cm section of petola was transferred into approximately 10–20 mL of liquid medium or plated onto a solid selective medium (agar thickness of 1 cm) in Petri dishes. Brine water samples were directly used for inoculation of selected growth medium. Each growth medium was designed to isolate different species of microalgae and cyanobacteria (see Supplementary Table S1—Culture_Medium_Contents): Johnson’s medium modified with 1.5 M NaCl [138], ASN-III medium [139], Artari medium [140], BG-11 medium supplemented with 0.6 M to 4 M NaCl [141], and Bold’s basal medium [142]. Following the initial inoculation, the cultures were incubated for 2–4 weeks at 22 ± 2 °C for the spring season and 26 ± 2 °C for the summer season under light intensity of 25 µmol m^−2^ s^−1^ ± 15% (cold white light). Cultures were observed regularly and subcultured into fresh medium once they turned dark green or reached high density. Enriched cultures were then purified in multiple steps using a combination of purification techniques, such as the streak plate method, serial dilution in liquid media and single cell isolation. For purification of some cyanobacterial strains, cultures were grown on 1% agarose plates filled with liquid medium to mimic natural conditions in salt pans and purified sequentially by transfer of single filaments.

### 4.5. DNA Barcoding: DNA Isolation, PCR and Sequencing of Laboratory-Grown Microalgae

Total genomic DNA was isolated from 5 to 10 mL of culture (the volume depended on the cell culture density). Microalgal biomass was pelleted by centrifugation and the pellet was resuspended in 500 µL of phosphate-buffered saline (PBS, pH 7.4 Thermo Fisher Scientific, Waltham, MA, USA). Next, samples were sonicated 4 times for 15 s (LABSONIC M ultrasonic homogeniser, Sartorius, Göttingen, Germany) on ice and centrifuged at 10,000× *g* for 10 min. The supernatant was used for extraction with DNeasy Blood and Tissue kit (Cat no. 69506, Qiagen, Hilden, Germany) according to the manufacturer’s instructions (starting at the 2nd step of the protocol). The elution volume was 100 µL. The concentration and purity of isolated DNA was determined spectrophotometrically (NanoDrop 2000c, Thermo Fisher Scientific, Waltham, MA, USA).

Internal transcribed spacer (ITS) genomic regions were amplified from isolated DNA by PCR Veriti Thermal Cycler (Thermo Fisher Scientific, Waltham, MA, USA). The ITS1-5.8S rDNA-ITS2 region of green algae was amplified using primers Fw_ITS1 and Rv_ITS4 [115]. For amplification of cyanobacterial ITS region, primers 322 and 340 were used [143]. PCR reactions were prepared as a 20 µL mixture, containing 12.4 µL of ultrapure water, 4 µL of 5× High-Fidelity green buffer, 0.2 mM dNTPs, 0.5 µM of primers, 1 µL of isolated genomic DNA (concentration 20–200 ng/µL), and 0.4 U of Phusion High-Fidelity DNA Polymerase (Thermo Fisher Scientific, Waltham, MA, USA). The PCR amplification protocol was as follows: initial denaturation at 95 °C for 8 min, 40 cycles at 95 °C for 30 s, Tm (see Table 6) for 45 s and 72 °C for 75 s, with a final extension step at 72 °C for 8 min.

The PCR-products were separated on a 1.5% (*w*/*v*) agarose gel in 1× TAE buffer and stained with ethidium bromide (0.5 µg/mL; Sigma-Aldrich, St. Louis, MO, USA). Single bands were excised and purified using the E.N.Z.A. gel extraction kit (Cat no. D2500-02, Omega Bio-Tek, Norcross, GA, USA), according to the manufacturer’s instructions. Purified PCR-products were directly sequenced by Sanger method (Eurofins Genomics, Ebersberg, Germany) using the primers described in Table 6.

Homologues of the algal (ITS1–5.8S rDNA–ITS2 and bacterial ITS) sequences were searched using the BLASTN heuristic algorithm for pairwise alignment against the core nucleotide (core nt) database. Match quality was evaluated based on sequence identity, coverage, and e-value [144].

### 4.6. Microscopy

Cultivated microorganisms were regularly observed and imaged with the optical microscope Primo star (Carl Zeiss, Microscopy GmbH, Jena, Germany). Observed morphological and physiological features included shape, length and width, color, motility, presence of multicellular structures, and the presence of extracellular mucilage (on filamentous cyanobacteria). Representative micrographs of cultivated cultures were taken using Plan-Achromat 100×/1.25 Oil objective (Carl Zeiss Microscopy GmbH, Jena, Germany).

## 5. Conclusions

This study is the first step in a comprehensive bioprospecting in the Sečovlje salt pans. We addressed the biodiversity of microorganisms, focusing on those with high potential for biotechnological applications and their remarkable ability to survive extreme environmental conditions. Furthermore, we established cultures of biotechnologically relevant green microalgae and cyanobacteria. The establishment of laboratory-scale cultures is significant for further optimization of biomass accumulation and therefore the yield of selected bioactive compounds. Among the targeted organisms, we established cultures of: *Tetradesmus obliquus*, which produces lutein, β-carotene, antioxidant compounds, and high fatty acid content; *Dunaliella* sp., which stands out for its high β-carotene and glycerol production; *Tetraselmis* sp., which produces polysaccharides, fatty acids such as EPA and DHA, and antimicrobial peptides; *Phormidium* sp./*Sodalinema stalli*; *Leptolyngbya* sp., which produces high protein content, UV-absorbing compounds (MAAs), phycocyanin, and polysaccharides; and *Capilliphycus guerandensis*, which has cytotoxic activity. Additionally, the metagenomic evaluation of biodiversity uncovered other organisms of potential biotechnological interest, such as *Halocynthiibacter arcticus*, *Spiribacter salinus*, *Cylindrotheca Closterium*, *Halomicrobium mukohataei*, and *Halopenitus persicus* (see the Supplementary Table S4_Extended_Discussion) [28,29,32,39,40,41,42,43,45,46,47,48,53,54,55,56,57,58,59,60,62,63,64,126,145,146,147,148,149,150,151,152,153,154,155,156,157,158,159,160,161,162,163,164,165,166,167,168,169,170].

It is important to highlight that a successful completion of laboratory-scale experiments does not guarantee the industrial transition. This transition requires more comprehensive and multidisciplinary research. The development of large-scale cultivation technologies, the reduction in contamination risk, and the increase in the yield of targeted metabolites are priority objectives. At the same time, the development of environmentally friendly, highly efficient, and cost-effective methods for the extraction and purification of products will determine the competitiveness of these natural compounds against their synthetic counterparts.

## Figures and Tables

**Table 1:** Species of biotechnological relevance that were present in the petola microbial mat at low-salinity environmental conditions.

Species	NCBI Taxonomy Superkingdom	Biotechnological Aspects	Sector of Application	References
*Dinoroseobacter shibae*	Bacteria	Production of poly-hydroxy-alkanoates (PHA)	Biomaterials (e.g., bio-based plastics)	[28]
*Halocynthiibacter arcticus*	Bacteria	Cold-active enzymes	Industrial processes	[29]
*Phaeobacter inhibens*	Bacteria	Production of tropodithietic acid and antimicrobial effect	Medicine	[30]
*Celeribacter manganoxidans*	Bacteria	Mn (II)-oxidizing	Environmental remediation	[31]
*Ruegeria* sp.	Bacteria	Production of Cyanophycin	Biomaterials (biopolymers)	[32]
*Thioclava nitratireducens*	Bacteria	Biofilm formation	Biomaterials	[33,34]
*Marivirga tractuosa*	Bacteria	Production of aldo-keto reductase	Cosmetics (retinol production)	[35]
*Roseobacter denitrificans*	Bacteria	Production of antibiotics and growth stimulants (auxins)	Aquaculture, medicine	[36]
*Leisingera methylohalidivorans*	Bacteria	Biofilm-forming capacity	Biomaterials	[37]
*Sulfitobacter pseudonitzschiae*	Bacteria	Bioluminescence, Biofilm formation	Industrial processes, biomaterials	[38]
*Fistulifera solaris*	Eukaryota	PUFAs and EPA production	Supplements and functional foods Pharmaceuticals	[39,40]
*Cylindrotheca closterium*	Eukaryota	Production of Diadinoxanthin and fucoxanthin	Food application and cosmeceutical applications	[41,42,43]
*Thalassiosira oceanica*	Eukaryota	Antimicrobial property and Bio-silica production	Biomedical Application	[44]

PHA, Production of poly-hydroxy-alkanoates; Mn, Manganese; PUFAs, polyunsaturated fatty acids; EPA, eicosapentaenoic acid.

**Table 2:** Species of biotechnological relevance that were present in the petola microbial mat at high-salinity environmental conditions.

Species	NCBI Taxonomy Kingdom	Biotechnological Aspects	Sector of Application	References
*Spiribacter salinus*	Bacteria	Ectoin and trehalose production	Industrial biotechnology (stabilizing)	[45,46,47,48]
*Haliscomenobacter hydrossis*	Bacteria	Hyaluronate lyase activity	Industrial biotechnology (hyaluronan processing)	[49]
*Haloferax gibbonsii*	Archaea	Production of extracellular polysaccharides	Industrial biotechnology (emulsification, surfactants)	[50]
*Haloquadratum walsbyi*	Archaea	Production of halomucin, archaeal gas vesicles	Medicine (protection against desiccation, vaccine development)	[51,52]
*Halomicrobium mukohataei*	Archaea	Intracellular silver nanoparticle production by exposure to silver, pigments	Medicine (antimicrobial, anticancer), cosmetics (antioxidant)	[53,54,55]
*Halopenitus persicus*	Archaea	Hydrolytic enzymes and carotenoids	Medicine (anticancer)	[56]
*Halogeometricum borinquense*	Archaea	Production of polyhydroxyalkanoates (PHAs)	Biomaterials	[57]
*Haloarcula hispanica*	Archaea	Production of PHAs, antioxidant effect	Biomaterials, medicine (anticancer)	[58,59]
*Haloferax volcanii*	Archaea	Halophilic enzymes, pigments, production of PHAs	Food and beverage industries, Medicine (antioxidant), biomaterials	[60]
*Halobiforma lacisalsi*	Archaea	Bacteriorhodopsin	Bioelectronics	[61]
*Natrinema* sp.	Archaea	Halocin, production of PHAs, chitinase	Medicine (anticancer), biomaterials, industrial biotechnology (chitin degradation)	[62,63,64]
Halorubrum trapanicum *	Archaea	β-Carotene production	Cosmetic	[65]
*Dunaliella salina*	Eukaryota	Β-Carotene production	Cosmetic	[66]

PHA, Production of poly-hydroxy-alkanoates; * is valid for *Halorubrum* sp.

**Table 3:** List of cultivated photosynthetic microorganisms isolated from Sečovlje salt pans. The table includes information about the final culture type (axenic or mixed), salinity conditions at the time of sampling (high salinity corresponds to 26.8–28.7% S—July and August, and low salinity corresponds to 4.4–5.1% S—April, May, September), sample types (petola or brine), and the growth media used for laboratory cultivation (freshwater medium—BBM; marine media—ASN-III, BG11+NaCl, BG11+Agar; hypersaline media—Modified Johnson medium, Artari medium). For each isolate, the corresponding GenBank accession numbers are listed.

Culture Type	Organism	Phylum, Order	Salinity	Sample Type	Growth Medium	GenBank Accession Number
Axenic and Mixed *	*Dunaliella polymorpha*	Chlorophyta, Chlamydomonadales	Low	Petola	Hypersaline	PX677330 *, PX677331, PX677332 *
Axenic	*Dunaliella* sp.	Chlorophyta, Chlamydomonadales	High	Brine	Hypersaline	PX677333
Mixed	*Dunaliella* spp.	Chlorophyta, Chlamydomonadales	Low and High	Petola and brine	Hypersaline	PX677334, PX677335, PX677336, PX677337, PX677338
Mixed	*Tetraselmis* sp. + unknown	Chlorophyta, Chlorodendrales + unknown	Low	Petola	Marine	PX684463
Axenic	*Tetradesmus obliquus*	Chlorophyta, Sphaeropleales	Low	Brine	Freshwater	PX684464
Axenic	*Phormidium* sp./*Sodalinema stali*	Cyanophyta, Oscillatoriales	Low and High	Petola and brine	Marine and Hypersaline	PX684465
Mixed	*Leptolyngbya* sp. + Prochlorotrichaceae bacterium	Cyanophyta, Leptolyngbyales + Prochlorotrichaceae	High	Brine	Hypersaline	PX684466, PX684467, PX684468, PX684469
Mixed	*Capilliphycus guerandensis* + unknown	Cyanophyta, Oscillatoriales + unknown	Low	Petola	Marine	PX684470

* These GenBank accession numbers correspond to sequences derived from mixed cultures.

**Table 4:** Biotechnological potentials, industrial applications, and market value of cultivated microalgae and cyanobacteria.

Species	Biotechnological Potentials	Industrial Applications	Market Value	References
*Tetradesmus obliquus*	Carotenoids (mainly composed of lutein, with β-carotene as a minor component), antioxidant compounds, high fatty acid composition	Food production and processing industry, Animal feed industry, Nutraceutical and functional foods industry, Pharmaceutical and biomedical industry, Cosmetics and cosmeceuticals industry	Β-Carotenoid: to reach USD 1.38 billion by 2030 Lutein: to reach USD 527.2 million by 2030 Astaxanthin: to reach USD 3.89 billion by 2030 Natural Antioxidants: to reach USD 1.50 billion by 2030	[90,91,92,93,94,95]
*Dunaliella polymorpha/Dunaliella* sp.	High β-carotene, glycerol, antioxidant pigments		Glycerol: to reach USD 5.67 billion by 2030	[96,97]
*Tetraselmis* sp.	Polysaccharides, fatty acids (EPA, DHA), antimicrobial peptides (AMPs)		Peptide Drug Conjugates: to reach USD 12,842.9 million by 2030	[98,99,100]
*Phormidium* sp./*Sodalinema stali*	Extracellular polysaccharides (EPS), phycocyanin, antimicrobial compounds Fatty Acid composition		Phycocyanin: to reach USD 276.4 million by 2030	[101,102,103,104]
*Leptolyngbya* sp.	Extracellular polysaccharides (EPS), UV-absorbing compounds (MAAs), phycocyanin High protein content		Sun care products: to reach USD 15.92 billion by 2030 Protein supplements: to reach USD 10.8 billion by 2030	[105,106,107,108]
Capilliphycus guerandensis *	Cytotoxic activity			[109]

* is valid for *Capilliphycus* sp.

**Table 5:** Information of samples collected from Sečovlje salt pans in 2023.

Month	Sampling Season	Water Temperature (°C)	Salinity (Mass %)/Salinity Level Category	Sample ID	Nanodrop Concentration [ng/μL] and Purity (A260/280 Ratio) of Replicates Passing Quality Control
April	Spring	20	4.4%/Low	April_1	144 (1.86)
				April_2	129 (1.85)
				April_3	99 (1.85)
				April_4	10 (1.83)
May	Spring	21	4.5%/Low	May_1	145 (1.85)
				May_2	65 (1.85)
				May_3	96 (1.84)
				May_4	118 (1.83)
July	Summer	33	26.8%/High	July_1	33 (1.78)
				July_2	31 (1.89)
				July_3	43 (1.95)
				July_4	130 (1.85)
August	Summer	32	28.7%/High	August_1	50 (1.91)
				August_2	101 (1.83)
				August_3	79 (1.84)
				August_4	155 (1.87)
September	Autumn	32	5.1%/Low	September_1	104 (1.82)
				September_2	113 (1.84)
				September_3	142 (1.85)
				September_4	108 (1.82)
				September_5	194 (1.84)

**Table 6:** Primers, used for barcoding samples.

Name	Sequence	Tm (PCR)	Purpose	Reference
**Fw_ITS1**	5′-AGGAGAAGTCGTAACAAGGT-3′	56.5 °C	Barcoding of green algae	[115]
**Rv_ITS4**	5′-TCCTCCGCTTATTGATATGC-3′	56.5 °C	Barcoding of green algae	[115]
**322**	5′-TGTACACACCGCCCGTC-3′	59.5 °C	Barcoding of cyanobacteria	[143]
**340**	5′-CTCTGTGTGCCTAGGTATCC-3′	59.5 °C	Barcoding of cyanobacteria	[143]

## Data Availability

The datasets generated and analyzed during the current study are publicly accessible in the GitHub repository at: github.com/NIB-SI/AlKoSol, which has been archived on Zenodo (DOI: 10.5281/zenodo.17975011). The raw shotgun metagenomic sequencing data generated in this study are available in the NCBI Sequence Read Archive (SRA) under the project accession number PRJNA1225550. Supplementary Data supporting the findings presented in the manuscript, including all Supplementary Tables, are provided within the Supplementary Materials. No additional datasets were generated or utilized beyond those made available through the repository and Supplementary Files. The nucleotide sequences generated in this study have been deposited in the NCBI GenBank database under the following accession numbers: PX677330, PX677331, PX677332, PX677333, PX677334, PX677335, PX677336, PX677337, PX677338, PX684463, PX684464, PX684465, PX684466, PX684467, PX684468, PX684469, and PX684470.

## Supplementary Materials

The following supporting information can be downloaded at: https://www.mdpi.com/article/10.3390/md24010026/s1, Supplementary Table S1—Culture_Medium_Contents; Supplementary Table S2—Petola species read classification; Supplementary Table S3—merged_results_Brine_salinity_factor; Supplementary Table S4—Extended_Discussion.

## Abbreviations

The following abbreviations are used in this manuscript:

PUFAs	Polyunsaturated Fatty Acids
FAO	Food and Agriculture Organization
USD	United State Dollar
EPA	Eicosapentaenoic Acid
CO_2_	Carbon Dioxide
pH	Potential of Hydrogen
MDS	Multidimensional scaling
LogFC	Logarithm of Fold Change
PHA	Production of poly-hydroxy-alkanoates
Mn	Manganese
β-Carotene	Beta Carotene
DNA	Deoxyribonucleic Acid
rDNA	Ribosomal Deoxyribonucleic Acid
ITS	Internal transcribed spacer
Be°	Baumé
PCR	Polymerase chain reaction
AGE	Agarose Gel Electrophoresis
MAE	Microwave-assisted extraction
SPE	Supercritical fluid extraction
UAE	Ultrasound-assisted Extraction
UV	Ultra Violet
DHA	Dihydroxyacetone
AMPs	Antimicrobial Peptides
EPSs	Extracellular Polymeric Substances
MAAs	Mycosporine-like Amino Acids
% S	Salinity
ng	Nanogram
μL	Micro liter
MaAslin2	Microbiome Multivariable Association with Linear Models 2
LM	Linear model fit
NEGBIN	Negative binomial
CSS	Cumulative sum scaling
TMM	Trimmed mean of M-values
BG11	Blue-Green medium 11
ASN-III	Artificial Seawater Medium III
µmol	Micromole
m	Meter
s	Second
mM	Millimolar
dNTPs	Deoxynucleotide Triphosphate
Tm	Melting Temperature
U	Unit
(*w*/*v*)	Weight/volume
TAE buffer	Tris–Acetate–EDTA
BLASTN	Basic Local Alignment Search Tool
