# Supplementary material for: Identification and Cultivation of Biotechnologically Relevant Microalgal and Cyanobacterial Species Isolated from Sečovlje Salt Pans, Slovenia
Source: Mar Drugs. 2026 Jan 8;24(1):26. doi: 10.3390/md24010026 (PMC12842852; doi:10.3390/md24010026)
Supplement: Supplementary file 1 [file marinedrugs-24-00026-s001.zip › Supplementary Table S4--Extended_Discussion_AR.docx]

***Supplementary Table S4—Extended Discussion***

***Non-targeted organisms with biotechnological potential: Identification of species of interest for future exploitation***

One of the aims of this study was to assess the diversity of hypersaline habitats with the aim to discover potential target species for future biotechnological exploitation. The next step in the bioprospecting pipeline for these organisms would be to selectively cultivate them and optimize the protocols for extraction of their compounds of interest. The compounds summarized in the table below are biomolecules identified in the Sečovlje salt pans microbiome through our metagenomic analyses, which are currently attracting significant interest in both academic and industrial circles. Particularly important for the cosmetics industry, some of these compounds have already been scaled up to production level and hold millions of dollars in economic value in the global market (see table below).

**Supplementary Table S4.** Some metabolites identified in metagenomic data obtained from the Sečovlje Salt pans that are important from a biotechnological perspective.

| **Product/Compound** | | **Species** | **Extraction Method** | **Market Value** | **References** |
| --- | --- | --- | --- | --- | --- |
| Production of polyhydroxyalkanoates (PHA) | - *Dinoroseobacter shibae* - *Haloferax volcanii* - *Natrinema* sp. - *Halogeometricum borinquense* - *Haloarcula hispanica* | | - Chemical extraction (solvents) - Mechanical disruption - Biological | estimated at USD 650.7 million/2023 and is projected to reach USD 1,222.1 million by 2030 | [28,57-60,62-64,126,145] |
| Cold-active enzymes | - *Halocynthiibacter arcticus* | | - Recombinant production - Cell disruption (mechanic, physical, chemical) - Purification | US$9.9 billion/2019 and is expected to grow at a rate of 7.1% between 2020 and 2027 | [29, 146-148] |
| Ectoin | - *Spiribacter salinus* | | - Bacteria milking - Cell disruption | grow to almost USD 0.12 billion by 2034 | [45-48, 149-151] |
| Trehalose | - *Spiribacter salinus* | | - Enzymatic production - Fermentative production - Chemical synthesis | To reach USD 663.63 million by 2032 | [45-48, 152-155] |
| Carotenoids (β-Carotene, Fucoxanthin, Diadinoxanthin) | - *Cylindrotheca closterium* - *Halomicrobium mukohataei* - *Halopenitus persicus* - *Haloferax volcanii* - *Halorubrum* sp*.* | | - Soxhlet extraction - Green Extraction - Microwave-assisted extraction (MAE) - Enzyme Extraction - Supercritical fluid extraction (SFE) - Pulsed electric field assessment extraction - Ultrasound-assisted Extraction (UAE) | Β-Carotene: to reach USD 952.5 Million by 2034,  Fucoxanthin:  To reach nearly USD 324.92 Million by 2032 | [41-43,53-56, 60,156-162] |
| PUFA s | - *Fistulifera solaris* | | - Fermentation production - Organic solvent extracton - Supercritical fluid extraction (SFE) | To grow by over USD 4.7 billion between 2025 and 2035 | [39-40,163-166] |
| Cyanophycin | - *Ruegeria* sp. | | - Solvent Extraction |  | [32,167] |

For example, bioplastics such as PHA (polyhydroxyalkanoates) are used not only in the production of environmentally friendly biomaterials but also in medical and cosmetic packaging solutions. The market size is projected to double by 2030, highlighting the strategic importance of this compound [168]. Cold-active enzymes offer innovative solutions in sensitive cosmetic formulations (e.g., anti-aging and sensitive skin products) because they can exhibit high catalytic activity at low temperatures [169]. Ectoin and trehalose are molecules that protect cells against stress conditions, particularly due to their moisturizing, anti-aging, and UV-protective effects [170]. The presence of producers of these compounds in our metagenomic data demonstrates the direct applicability of halophilic microorganisms' adaptation strategies to extreme conditions in industry.

Overall, this picture demonstrates that the extreme microbial communities of the Sečovlje salt pans are valuable not only ecologically but also biotechnologically and economically. The data obtained provides a strong foundation for the integration of local biodiversity into sustainable biotechnology.

*Challenges*

1. Biodiversity evaluation. Accurate and sensitive biodiversity analysis of samples taken from their natural environment is essential as studies based only on estimates and assumptions may overlook potentially (ecologically or biotechnologically) relevant microorganisms. It is also important to consider how the rapidly changing abiotic conditions throughout the year affect the distribution of species. Such studies are time-consuming and costly. Hence, sampling campaigns (either supported by national monitoring campaigns or funded long-term monitoring through projects) should be conducted at regular intervals to uncover geographic and temporal microbial diversity. These campaigns should include metagenomics assessment on biodiversity to determine the diversity of species prior to culturing and isolation of targeted species;
2. Culturing. Simulating the laboratory environment and sustaining the behavior of organisms in their natural environment is a critical step to obtain locally sourced cultures of biotechnological relevance. However, by focusing only on the establishment of pure cultures, symbiotic and competing relationships in natural environments are often overlooked or unknown, and symbiotic and competing organisms in the natural environment may be absent when pure cultures (axenic) are grown in laboratory conditions. Indeed, co-culturing can often trigger or enhance the production of desired bioactive compounds . On the other hand, working with co-culture or a mixture of cultures increases the complexity and fine-tuning needs to optimize the cultivation conditions. Nevertheless, to capture the biotechnological potential of microorganisms (in axenic or mixed cultures), different media and isolation techniques should be used to optimize the production of target compounds;
3. Extremophiles. Besides, their under valorized biotechnological potential, they are also good candidates for large-scale production due to reduced needs for maintaining sterile conditions under their extremophilic metabolic requirements, which facilitates their production in open bioreactors. However, the slow growth rates of these microorganisms infer delayed treatment processes, while creating mandatory energy costs for maintaining the correct environmental parameters .Therefore, it is desirable to create milder conditions for selected extremophilic strains to reduce production costs. But by modifying the culture medium or environmental conditions can affect the composition of micro-algal biomass and make the process inappropriate. Therefore, before scaling up the processes into industrially relevant quantities, any modifications of physical and chemical parameters should be done prior to the actual scaling up to assess whether adaptation processes change the biochemical composition of microbial biomass.
